# Stem cell sources and characterization in the development of cell-based products for treating retinal disease: An NEI Town Hall report

**DOI:** 10.1186/s13287-023-03282-y

**Published:** 2023-03-29

**Authors:** Ashley M. Fortress, Kiyoharu J. Miyagishima, Amberlynn A. Reed, Sally Temple, Dennis O. Clegg, Budd A. Tucker, Timothy A. Blenkinsop, George Harb, Thomas N. Greenwell, Tenneille E. Ludwig, Kapil Bharti

**Affiliations:** 1 National Eye Institute, National Institutes of Health, Bethesda, MD USA; 2 Retinal Neurophysiology Section, National Eye Institute, NIH, Bethesda, MD USA; 3 Neural Stem Cell Institute, Rensselaer, NY USA; 4 Center for Stem Cell Biology and Engineering, University of California, Santa Barbara, CA USA; 5 Institute for Vision Research, Department of Ophthalmology and Visual Science, Carver College of Medicine, University of Iowa, Iowa City, IA USA; 6 Ophthalmology Cell Development and Regenerative Biology, Black Family Stem Cell Institute, Icahn School of Medicine at Mount Sinai, New York, NY USA; 7 Cellino Biotech, Cambridge, MA USA; 8 WiCell Research Institute, Madison, WI USA; 9 Ocular and Stem Cell Translational Research, National Eye Institute, NIH, Bethesda, MD USA

**Keywords:** Embryonic stem cells, Induced pluripotent stem cells, Clinical manufacturing, Cell replacement therapy, Retinal degeneration, Autologous, Allogeneic, Translational research, Cell sources, Cell characterization

## Abstract

National Eye Institute recently issued a new *Strategic Plan* outlining priority research areas for the next 5 years. Starting cell source for deriving stem cell lines is as an area with gaps and opportunities for making progress in regenerative medicine, a key area of emphasis within the NEI *Strategic Plan*. There is a critical need to understand how starting cell source affects the cell therapy product and what specific manufacturing capabilities and quality control standards are required for autologous vs allogeneic stem cell sources. With the goal of addressing some of these questions, in discussion with the community-at-large, NEI hosted a Town Hall at the Association for Research in Vision and Ophthalmology annual meeting in May 2022. This session leveraged recent clinical advances in autologous and allogeneic RPE replacement strategies to develop guidance for upcoming cell therapies for photoreceptors, retinal ganglion cells, and other ocular cell types. Our focus on stem cell-based therapies for RPE underscores the relatively advanced stage of RPE cell therapies to patients with several ongoing clinical trials. Thus, this workshop encouraged lessons learned from the RPE field to help accelerate progress in developing stem cell-based therapies in other ocular tissues. This report provides a synthesis of the key points discussed at the Town Hall and highlights needs and opportunities in ocular regenerative medicine.

## Introduction

For the first time in over 50 years, the National Eye Institute (NEI) has issued a new mission statement, which is “To eliminate vision loss and improve quality of life through vision research.” Along with its newly minted mission statement, NEI released the *National Eye Institute Strategic Plan: Vision for the Future* in November 2021, which outlines its direction and priorities over the next 5 years [1, 2]. The Strategic Plan reflects emerging themes in eye and vision research and ultimately identifies research opportunities that could help improve population health in many fields of vision research. The goal of the NEI Strategic Plan is to remain focused on NEI’s mission while looking for opportunities to leverage innovation and activities of other ongoing initiatives.

Characterizing the differences between cell sources was identified as a topic within regenerative medicine that currently has gaps and opportunities for the vision community. Specifically, the regenerative medicine community recognizes the opportunities created by advances in stem cell research to produce new cells and tissues for transplantation to benefit patients suffering from blinding disorders. The community also recognizes the critical importance of standardization of procedures and protocols in the field of cell transplantation, and the necessity for rigorous comparison of different products as well as allogeneic and autologous cell approaches [3]. Moreover, there is still a need to understand which stem cell lines to use for generating a cell therapy product, how and when the cell therapy product is to be delivered to the patient, and how to track transplanted cells safely and accurately. Furthermore, generating safe, durable, specific, efficient, and scalable products for transplantation is an area of need presenting opportunities for innovation.

To address important, unanswered questions pertaining to stem cell sources, characterization of derived ocular cell types (e.g., photoreceptors, retinal pigment epithelium (RPE), and retinal ganglion cells), and preclinical replacement strategies, NEI hosted a Town Hall at the Association for Research in Vision and Ophthalmology (ARVO) annual meeting in May 2022. Leveraging recent research advances and regulatory success stories for RPE replacement strategies [4–6], the session discussed improvements in cellular product development and how to increase the likelihood of success in restoring sight using cellular therapies. We provide a synthesis of the key points from six topics presented from leading experts in the field and highlight current needs and opportunities for the future.

## Background

### Stem cell-based replacement therapy in retinal degenerative diseases

Current research indicates that we may soon be able to replace lost or damaged cells in the eye, restore function, and prevent vision loss from numerous degenerative eye diseases [4, 7, 8]. Retinal degenerative diseases are a category of blinding diseases that are characterized by a progressive loss of photoreceptor cells. Retinal degenerative diseases are a major cause of blindness resulting from inherited conditions or comorbidity with non-ocular conditions such as diabetes and aging. For example, age-related macular degeneration (AMD), a leading cause of vision loss among the elderly, is an incurable disease that results in dysfunction and death of the retinal pigment epithelium (RPE), a monolayer of specialized cells that are crucial for maintaining homeostasis of the outer retina. Annually, it is estimated that AMD affects 11 million people in the US and 196 million worldwide, and can greatly affect the quality of life of those afflicted [9]. Importantly, the retina cannot repair itself and restore vision once RPE or photoreceptor loss occurs. To this end, NEI established the Audacious Goals Initiative in 2013 to focus on restoring vision through photoreceptor and retinal ganglion cell replacement [10, 11]. This goal of cellular replacement is supported in part by the relative “immune privileged” state of the eye [12, 13], and small anatomical size requiring fewer transplanted cells [14]. Recent advances in single cell resolution retinal optical imaging also make it possible to noninvasively monitor disease progression and responses to cellular therapy [15, 16].

As advances have been made in both basic and translational regenerative medicine, the barriers to successful transplantation have evolved. Thus, cell sourcing and characterization remain fundamental to bringing safe and effective treatments to the clinic. Additional considerations include transplantation strategies for different ocular cell types; the role of the immune system in response to transplanted cells; the role of reprogramming in cell transplantation; the importance of quality control and validation of stem cells and derived ocular cell therapy products; and methods to increase production of cell products efficiently and effectively for scale.

### Principles of quality control standards in stem cell-based products

Cell sources are unquestionably important. The recipient (patient) of these therapies is also important in defining success (i.e., when to deliver cells, early or late-stage disease) and identifying the ideal window for treatment. Additionally, each recipient may respond differently to the same treatment because of the different immune competencies of each host [17, 18]. Overcoming these challenges will require a collaboration between researchers across disciplines, the leveraging of cellular technologies from industry to achieve scalability, and increased early interaction and engagement with regulatory authorities to advance cellular therapies to the clinic. Quality controls are essential both at the initial stem cell stage and in the final differentiated cell product stage. For instance, stem cells require evaluation for genomic stability, absence of potentially oncogenic mutations, loss of reprogramming constructs, and identity. For final products [19, 20], release testing ensures that the cells returned to the patient have appropriate composition (identity and purity) and are verified to be safe and effective (potent). In this regard, donor-derived allogeneic therapies have a manufacturing cost advantage as only a few donors will need to be screened and tested for potential latent infections, providing a cell bank for future treatments (“off the shelf”). While patient-derived autologous cell sources will each have to undergo testing of final product for each patient (“service based”). In both autologous and allogeneic scenarios, the final product testing includes purity of cells (absence of undifferentiated stem cells and presence of the desired markers for the desired cell, e.g., RPE markers in RPE transplants), cell viability, identity back to the donor, and functional tests (e.g., polarized secretion of cytokines and junctional intactness of the RPE monolayer) [7].

### Advantages of allogeneic or autologous stem cell sources

One of the biggest questions that remains in the field of cell transplantation is whether to use allogeneic or autologous stem cell sources for the derivation of cell therapy products. Allogeneic sources use donor-derived stem cells whose human leukocyte antigens (HLA) may or may not match the recipient’s or the donor stem cells. In a relatively new approach, allogeneic stem cells can be engineered to lack all HLA genes making them “cloaked” to the host immune system. In theory, a single donor could provide sight saving treatments to many patients. These include stem cells derived from blastocysts [21]—human embryonic stem cell (hESC) or induced pluripotent stem cells (iPSCs). hESC-derived (RPE) were the first pluripotent stem cell-derived RPE cells to be transplanted into patients [8]. Initial clinical trials were focused on evaluating safety and tolerability [22–26]. In contrast, autologous cell sources use a patient’s own cells, which are first cultured *ex-vivo*, expanded, reprogrammed into iPSCs, differentiated, and then returned to the same patient.

Both ESCs and iPSCs have the capacity to become any cell type and have unlimited proliferation potential but at the same time have the possibility of being tumorigenic and can have karyotype or genetic instability. For this reason, both allogeneic and autologous cell sources require testing to verify the identity of the cells and exclude chromosomal abnormalities (karyotyping). The regulatory requirement for cell products’ inability to transfer latent infection requirement is more stringent for allogeneic cells than autologous cells. This is because allogeneic cell products may put several patients at risk, whereas with autologous cells, the risk is to a single patient, making donor screening for latent infections a requirement for allogeneic stem cell-based therapies [27]. Partially developed “fetal-like” cells may harbor the potential of becoming unwanted cell types. Effort has been focused on making post-mitotic, fully mature RPE cells from RPE stem cells present in adult cadaver eyes [28], iPSCs [29], and ESCs [4], reducing the risk for tumorgenicity. A risk-based approach guides decisions on the stage of development at which the cells should be transplanted into the eye [30].

RPE cells have been delivered in either a suspension or on a scaffold (biostable or biodegradable). Delivery of cell suspensions requires a less invasive surgical procedure that is easier to heal—with the hope that the cells may naturally integrate into the existing RPE monolayer under the retina. Scaffolds allow RPE cells to be delivered on an implantable surface that keeps cells properly oriented (polarized) [6, 31], providing similar mechanical and diffusion properties to underlying tissue (e.g., Bruch’s membrane) and may improve cell survival in animal models [32]. However, surgical delivery of scaffolds can be challenging as it requires creating a localized retinal detachment followed by relatively large retinal incision [6, 28, 33–36]. Risks include subretinal bleeding, recurrent retinal detachment, and potential to develop proliferative vitreoretinopathy (PVR) [37], although new techniques are being developed to limit surgically induced trauma [6].

### Genetic modification and cellular reprogramming of stem cells for transplantation

Recent advances in gene modification provide opportunities to alter gene expression patterns and/or control cellular reprogramming in the retina. For example, solutions offered by genetic modification include non (or hypo)-immunogenic genetically engineered universal donor allogeneic cells [38, 39], and the ability to correct genetic mutations in autologous stem cells and then transplant the derived cell therapy back into the eye of the stem cell donor. One concern with gene modified cell therapy products is that there is a possibility of off-target genomic modifications and genomic instability, increasing the possibility of undesired products or transplants that may become cancerous. Furthermore, hypo-immunogenic (HLA-null) stem cell products may escape immune-surveillance if they become cancerous or become a viral “sink”. Because of these concerns, there is a higher regulatory burden for auditing of the genome in genetically modified cell therapy products. Analogous to the situation with latent infection transfer, this burden of genomic quality control is even higher for allogeneic stem cell banks—because derived products may be transplanted in a larger population. Due to a higher threshold for auditing, clinical translation of genetically modified products has been slow with no current ongoing trials in the eye field. Different reprogramming strategies have been developed over the years starting with Takahashi and Yamanaka in 2006 [40] and have evolved to include other vectors [6, 41–43] and non-viral strategies [44–47] to deliver reprogramming factors. Using well-defined chemicals [48], the transgene-free generation of human iPSCs is potentially more cost effective and regulatory compliant than viral methods.

### Immune considerations

The healthy eye is thought to be immune-privileged, meaning that the eye is protected from immune insults by the blood retina barrier. However, the blood retina barrier is often compromised in many retinal diseases leaving transplanted cells vulnerable to systemic immune responses. This poses a unique challenge for allogeneic cell survival after transplantation. Recent evidence suggests that transplanted HLA-mismatched RPE cells can survive two years after transplantation following an initial short course, low dose tacrolimus regimen [25]. In comparison, a 4-year follow-up of the single patient transplanted with an autologous iPSC-RPE graft also shows cell survival and support of photoreceptors and choroid [49]. Long-term follow-up with multiple patients in each category will be required to perform a comparative analysis of graft integration and survival. Furthermore, it is not clear if immune challenges faced by photoreceptor transplants will be similar to RPE cells. RPE cells are thought to be immune-suppressive in nature and may contribute to locally silencing the immune response [50, 51]. A great deal of factors must be weighed to optimize systemic immune suppression, including which drug combinations to use, the time course of delivery, and the ocular cell type transplanted [18]. Some researchers are testing whether it is beneficial to provide localized immunosuppression as this could help alleviate the concern that systemic immunosuppression may cause severe adverse events, especially in older patients [19]. Recently, stem cell-derived precursors of human photoreceptors were successfully delivered to canines and tracked over time using noninvasive imaging techniques. In dogs that had advanced retinal degeneration, introduced cells were able to integrate and connect to second order neurons. Using an immunosuppressive cocktail greatly extended long-term survival of these xenotransplants [52].

### Manufacturing and scalability challenges

Human ESC and iPSC culture methods are labor intensive making them difficult to scale up. Autologous and allogeneic therapies pose different challenges: while allogeneic stem cell banks need to be scaled up so they can be delivered to a large population, in the case of autologous cell therapies the manufacturing process needs to be scaled out for simultaneous manufacturing of cells for multiple patients. Current advances in automated cell culturing are making it possible to achieve commercial-scale manufacturing, while producing more consistent products and reducing contamination [53]. NEI recognizes that translating RPE, photoreceptor, and retinal ganglion cell replacements to clinical care is an ambitious goal but research advances in cellular therapies suggest we are closer to it than previously thought [4, 7, 8].

### Extracellular vesicles as potential therapeutic agent

Within the Strategic Plan, multiple priority areas and areas of emphasis were proposed including the role of extracellular vesicles (EVs) in ocular regeneration. EVs are membranous micro-vesicles secreted by cells that have been shown to contain proteins, RNA, even an intact organelle in some cases. Exosomes are a nanoscale subclass of EVs that haven been linked to disease induction and regeneration. For instance, mesenchymal stem cell-derived EVs have been demonstrated to protect ocular cells from degenerative and inflammatory conditions [54–57]. A detailed discussion about EVs and their regenerative capacity is beyond the scope of this White Paper but EVs are thought to have enormous therapeutic potential.

## ARVO Town Hall

This ARVO Town Hall was co-chaired by Dr. Kapil Bharti, Senior Investigator from NEI, and Dr. Tenneille Ludwig, Director of WiCell Stem Cell Bank. Subject matter experts were identified to speak on six topics central to the goal of this event. Dr. Michael Chiang, NEI Director, kicked off this meeting by giving an update on Strategic Planning efforts and Regenerative Medicine activities at the NEI. The Town Hall continued with each of the invited subject matter experts speaking on topics (identified below) that are important for cell sources and cell characterization for transplantation. The session concluded with a moderated discussion by Dr. Kapil Bharti.

### Dr. Sally Temple: “Tissue-derived and pluripotent stem cell-derived cells for retinal repair”

Stem cells are present at all stages of development. At the early stage when the embryo consists of a hollow fluid-filled sphere termed a blastocyst, a small collection of cells in the interior of the sphere called the inner cell mass can be grown in tissue culture in conditions that maintain the cells as self-renewing ESCs. ESCs are pluripotent, i.e., they can give rise to any somatic cell type. Later in development, at the fetal stage, stem cells are involved in producing different tissues and organs in the body. These tissue-derived stem cells are not pluripotent but are restricted to producing a smaller repertoire of progeny, typically related to the tissue in which the stem cell resides. Once development is complete, some tissues and organs including most brain regions and neural retina do not have many remaining stem cells [58–61], while others, such as skin [62], bone marrow [63] and muscle [64], retain a stem cell population throughout life. These adult stem cells help regenerate and repair their respective tissues, for example, muscle satellite cells are activated upon muscle injury to divide and produce new muscle fibers and bone marrow hematopoietic stem cells continually replenish the diverse population of blood cells. Extensive research has shown that populations of adult stem cells can be restricted in their potency: for example, hematopoietic stem cells will typically not produce brain cells [65] and myogenic satellite cells will not produce blood cells, unless they are genetically altered to do so, e.g., through reprograming methods. On the contrary, distinct populations of multipotent adult stem cells have also been documented in such tissues such as muscle and their cell fate determination have been shown to be driven in a context-specific manner influenced by factors or signals provided by the host microenvironment [66]. Hence, for tissues that maintain stem cells into adulthood, many can be repaired with appropriate stem cells and immunosuppressive therapies as needed. However, as mentioned, resident populations of stem cells may be rare or dormant in several tissues, and for these tissues, pluripotent stem cells offer an exciting opportunity for tissue repair.

Additionally, pluripotent stem cells as an alternative cell source can be successfully differentiated into neural retina and into RPE cells, offering the promise of regenerative cell replacement for degenerative retinal diseases. Indeed, several pluripotent stem cell-derived RPE products are already in clinical trials. Because pluripotent stem cells are highly proliferative and tumorigenic if injected in sufficient number, it is necessary to ensure that pluripotent stem cells or highly prolific progeny are essentially removed from the cell product prior to transplantation to avoid tumor formation or other unwanted products post-transplantation. Regulatory guidelines for clinical use of pluripotent stem cells are available [67, 68] and being further developed by stakeholder communities such as the International Society for Stem Cell Research.

For RPE cell replacement, in addition to pluripotent stem cell sources, researchers can utilize an adult stem cell. In 2012 we described an adult RPE stem cell (the RPESC) present throughout life, even in patients in their 90s [69]. We demonstrated that adult RPESCs can be dramatically expanded in vitro to create cells with key physiological characteristics of the native RPE layer [70] and sufficient cells for hundreds of doses. Importantly, they can be transplanted successfully as a mature RPE monolayer in animal models [6, 28]. In addition, they can be transplanted as a suspension of intermediate progenitor cells that are post-mitotic but not fully differentiated; after subretinal injection into the RCS rat of retinal degeneration, adult RPESC-derived RPE progenitor cells prevented photoreceptor and vision loss [71, 72]. These adult RPESC-derived cells could successfully integrate into the existing RPE layer and persist long-term in animal models without adverse safety findings, supporting their use as RPE cell replacement therapy. Adult RPESC-RPE cells in suspension are currently in clinical trial as allogeneic therapy for patients with dry AMD [73].

With several RPE transplant therapies underway, additional efforts are focused on repairing the neural retina. Because pluripotent stem cells can efficiently produce photoreceptors and their progenitors in vitro, these stem cells are the primary source of photoreceptor cells being developed for diseases such as retinitis pigmentosa. Current hurdles include efficient cell replacement and integration into existing circuits, problems that must also be overcome for replacement of other retinal cells such as retinal ganglion cells. Transplantation of pluripotent stem cell-derived photoreceptors and other neural retinal cells is still in preclinical phases [52, 74–77]. An alternative source of neural retinal cells comes from fetal human eyes, and trials using these cells for patients with retinitis pigmentosa are being pursued. ReNeuron has injected human fetal retinal progenitor cells subretinally in a study that the company is no longer pursuing due to limited efficacy and surgical complications that in some cases caused reduced vision. JCyte is also using human fetal retinal progenitor cells, but rather than injecting them subretinally, their approach entails injecting them into the vitreous in order to provide additional trophic support, with the goal of slowing the demise of affected photoreceptors [78].

Each of the above-mentioned efforts and clinical trials are being performed under regulatory oversight that requires stringent cell manufacture and strong evidence supporting an investigational new drug (IND) application to the FDA in the USA or equivalent regulatory oversight bodies in other regions worldwide. The clinical trials themselves are carefully monitored by independent bodies. However, it is important to point out that some nefarious operations are capitalizing on the promise of stem cell research by setting up so-called clinics claiming to offer treatments for these blinding disorders that are done without regulatory oversight [79]. In some cases, the clinic performs a liposuction then injects the mixture into the eye claiming that the mixture contains stem cells that can benefit the patient’s vision [80]. Horrifically, several individuals have been blinded [80]. In addition, the growing demand for cell therapies has led to “transplant tourism” whereby patients undergo stem cell treatments in countries with relaxed regulatory oversight [81–84]. Unethical practices including internet-based direct to consumer advertisements of unproven treatments [85] have led to government attempts to implement changes [86]; however, it remains unclear whether the infrastructure needed to completely block such unwarranted “treatments” will be available and if such efforts will be successful [87, 88].

Thus, to protect patients from these nefarious practices we must provide education about genuine efforts in regenerative medicine that are being conducted with appropriate oversight, in a carefully regulated manner, with patient welfare at the center. For example, the International Society for Stem Cell Research supports an online resource for patients to help evaluate stem cell treatments and identify misinformation and unproven treatments [89].

### Dr. Tenneille Ludwig: “QC and CQA: quality standards for translational work”

Establishing and maintaining quality in material throughout the manufacturing process is critical to the success of any translational work. Even the best science and technology can be completely undermined if the underlying quality of materials is compromised. While differences in target and context will prevent a “one size fits all” approach to essential quality control (QC) and critical quality attributes (CQAs), there are some concerns common to all cultures that should be acknowledged and addressed in any QC program. These primarily concern necessary testing to assure basic material authenticity, quality, and safety.

For allogeneic cell therapies, two-tier banking systems are recommended (Master cell and Working Cell Banks) with QC testing to assure both the quality and safety of materials. Testing should include at minimum:

- Viability, recovery, and morphology to assure the ability to recover a normal, apparently healthy culture.
- Authentication of identity to guard against any misidentification or cross-contamination of cultures.
- Genomic assessment including interrogation of the whole genome (e.g., karyotype, whole genome sequencing) and targeted regions (e.g., p53) to confirm expected genotype.
- Blood type and histocompatibility.
- Sterility including bacteria, fungi, and mycoplasma status to assure cultures are free from contamination that may impact the health and function of the resulting cells.
- Human and animal pathogens as appropriate to protect both the research team and patients. For example, a major concern potentially limiting cellular therapeutic use across international borders is related to prion diseases such as Bovine Spongiform Encephalopathy and Creutzfeldt–Jakob Disease because there is no regulated test. For this reason, in the US, the FDA mandates not using bovine or donor-derived products from European Union (EU) or United Kingdom (UK) manufacturers.

The specific assay selected to assess these areas should be chosen to meet the specific regulatory requirements of the jurisdiction of the intended trial. It is important to recognize that while most regulatory agencies require similar testing, there are subtle differences in specific requirements that may require additional testing for approval across jurisdictions [68, 90]. Being aware of these differences up front may save considerable difficulties down the road.

Where to go for more information on testing regulations:

- In the US
  - US FDA21 CFR1271 (Subparts C & D) and ICH Q5A(R1) and Q5D (https://www.accessdata.fda.gov/scripts/cdrh/cfdocs/cfcfr/CFRSearch.cfm?CFRPart=1271)
- In the EU
  - European Union Tissue and Cells Directives; Annex II Directive 2006/17/EC. (https://www.legislation.gov.uk/eudr/2006/17/annexes)

### Dr. Dennis Clegg: “Cellular therapies for eye disease: allogeneic vs autologous cell sources”

Many studies support the concept of partial immune privilege within the eye. Contributing factors are thought to include: the blood-ocular barrier, anti-inflammatory and tolerogenic microenvironments, and what has been called the anterior chamber associated immunity deviation, where suppression of effector T cells and induction of T regulatory cells may occur via secreted factors [91, 92]. However, there can be breaches of this partial immune privilege due to damage to the blood-ocular barrier, uveitis, immune rejection of corneal grafts, or rejection of transplanted RPE and photoreceptors [93].

As discussed above, both allogeneic and autologous approaches are currently being advanced for transplantation of RPE cells for the treatment of age-related macular degeneration. Some promising results have emerged from clinical trials utilizing injection of cell suspensions or implantation of RPE monolayers on some type of scaffold [4, 25, 26], but it is too early to tell which approach will prove to be the most effective, and how long cells will survive after transplantation into a potentially toxic microenvironment. A recent study showed that unmatched allogeneic hESC-RPE cells survived after two years in a patient with severe geographic atrophy [94]. This patient was given a short course of systemic immunosuppression before and after the surgery, suggesting that immunosuppression may contribute to long-term survival of transplanted cells. It has also been shown that in the absence of immune suppression, the rejection of transplanted allogenic iPSC-derived RPE cells is quite high [95].

### Dr. Timothy Blenkinsop: “Hypoimmune platform and immune considerations for transplantation”

A major stumbling block for research teams optimizing immunosuppression regimens is partially due to the diverse immune systems sensitivities of the model systems used for optimizing cell transplantation. From tree shrews to ground squirrels to rodents, pigs and monkeys, each species has a unique response to cell transplantations and requires a tailored immunosuppression regimen [6, 18, 72, 96–98]. This diversity means each transplant team must develop two immunosuppression approaches, one for the animal in which they test cell transplantation approaches and one for humans when they reach the clinic, which has slowed progress substantially.

Nevertheless, there is optimism as lessons have been learned from varying areas of study. Heart and lung transplantation fields have developed immunosuppression regimens; the most common being a maintenance immunosuppression (M-IMS) regimen consisting of tacrolimus, mycophenolate mofetil (MMF), and corticosteroids (temporarily) [99]. In heart transplantation the 3-year patient survival rate was found to be 97% for a regimen consisting of tacrolimus (a calcineurin inhibitor) and mycophenolic acid (MPA) (an antimetabolite), and in lung transplantation the 3-year patient survival rate was 75% for MMF in combination with anti-thymocyte globulin (ATG) induction, cyclosporine (a calcineurin inhibitor), and corticosteroids [99]. Inhibition of toll-like receptors and other immune recognition genes associated with CD4, CD8, and natural killer cell engagement have demonstrated success in animal models but have yet to be tested in the clinic [100]. Suppression of MHC classes have also demonstrated some success in vitro but has yet to prevent host attack in vivo [101]. This is likely due to natural killer cells detecting the absence of MHC presenting antigens thereby targeting cells for attack [102, 103]. Recent studies have shown that under inflammatory conditions double knockout of HLA-I and HLA-II in hESC-RPE results in increased activation of the recipient’s innate natural killer cells in a donor-dependent manner [38]. This suggests it may be possible to screen donor hESC-RPE lines in order to minimize reactivity with the recipient NK cells helping to avoid eliciting a cytotoxic response.

Additional approaches at mitigating graft immune attack consist of testing immunosuppression compounds on host lymphocytes ahead of transplant to validate efficacy [18]. Recent work transplanting adult human RPE on porous polyester terephthalate demonstrated survival under the macaque fovea after three months using mTOR inhibitor one-week prior to transplantation and for the duration of the experiment [28]. This evidence is in addition to the previously discussed example where the transplant lasted two years in a patient receiving temporary immunosuppression before and after transplantation procedure [94]. We are also beginning to understand the varying transcriptional states of RPE important for maintaining cell stability [104]. Together, these studies point to the field homing in on an effective surgical and immunosuppression approach that will lead to long lasting functional replacement.

### Dr. Budd Tucker: “Autologous photoreceptor cell replacement”

Autologous iPSC-mediated photoreceptor cell replacement is one of the most promising therapies currently being developed for patients suffering from inherited retinal degenerative blindness. For those attempting widespread application of this technology, the following are important considerations. First, the genetic integrity of donor iPSCs at all stages post-reprogramming, clonal expansion and CRISPR correction must be ensured. Second, the manufacturing strategy used to generate the clinical product must be designed to enable parallel production (i.e., simultaneous generation of transplantable cells from multiple patients). In this section, we will discuss some of the most common issues associated with clinical manufacturing of autologous iPSCs and how novel robotic platforms can be implemented to support clinical translation.

#### Ensuring genetic integrity of the clinical product

Several recurrent genetic abnormalities have been reported in both embryonic and induced pluripotent stem cells, including duplication of chromosomes 12 and 17 [105], copy number variations at 1q31.3 and 17q21.1 [106], and dominant negative mutations in the tumor suppressor gene P53 [107]. As chromosomes 1, 12 and 17 contain several cell cycle genes, duplication was found to confer a selective growth advantage, as such their frequency increased with extended passage (e.g., ESCs that had a normal karyotype at passage 45 acquired complete trisomy of chromosome 12 by passage 63). While less common in early passage cells, trisomy 12 has been detected in iPSC cultures as early as passage 14, which may result from the uniquely high selection pressure exerted during colonial expansion [105].

For autologous photoreceptor cell replacement in patients with inherited retinal diseases, genetic correction of the patient’s iPSCs prior to differentiation will likely be required. CRISPR mediated genome editing is one of the most promising and tractable technologies available for this purpose. The CRISPR system relies on the use of small guide RNAs to target a nuclease, which is capable of inducing DNA double-stranded breaks, to a desired location within the cells genome. Double-stranded breaks are subsequently repaired via non-homologous end joining, which is imperfect resulting in formation of indels, or precise template-mediated homology directed repair [108]. Using the CRISPR system we have demonstrated successful correction of exonic, deep intronic and dominant gain of function mutations within patient iPSCs that range from single base pair changes to large insertions and deletions [109–111]. One drawback of the CRISPR technology, which is true of any genome editing approach, is the potential for deleterious unintended editing. For instance, large on-target mono-allelic genomic deletions resulting in loss-of-heterozygosity that are difficult to detect using standard PCR and Sanger sequencing have been reported in CRISPR edited iPSCs [112, 113]. Similarly, we have demonstrated off-target-edits in intronic and intergenic space [109–111]. While most unintentional edits are non-functional, in-depth genetic analysis of iPSC lines following CRISPR correction is required.

To detect genetic integrity issues within donor iPSCs, stringent release testing must be implemented early within the production pipeline. It is paramount that this testing be performed sufficiently close to differentiation to ensure that issues have not arisen in the interim. For instance, we have opted to perform CRISPR correction between passage 4 and passage 8 when iPSCs are in the early stage of clonal expansion. We evaluate each clone at passage 10 using the hPSC Scorecard™ Panel, karyotyping, and whole genome sequencing at a minimum sequencing depth of 50 million reads, to demonstrate that cells are devoid of reprogramming factors, pluripotent, have normal chromosomal structure, and lack mutations in retinal, cell cycle, and known cancer genes. The resulting validated cells are subsequently banked, and differentiation is initiated at passages 12–14. While validated iPSCs are rarely used beyond passage 18 in our pipeline, if the parent iPSC line is maintained, the above release testing strategy is repeated at passage 20 and again at least every ten passages thereafter.

#### Clinical manufacturing and parallel processing

The greatest strength of the iPSC technology is the ability to generate therapeutic cells from the patient for whom they are intended (i.e., autologous). Great effort has gone into development of manufacturing technologies that enable scale-up and large batch production of cellular therapeutics intended for the treatment of large patient populations. While excellent for allogeneic approaches, this scale-up strategy is not well suited for production of autologous, patient specific therapies. For autologous cell replacement to be clinically relevant parallel small batch production of individualized products is required. Using current cell reprograming and retinal differentiation protocols, it takes months to generate CRISPR-corrected transplantable photoreceptor cells. As such, a single scientist can make products for just a handful of patients per year. Scale-up using manual cell culture approaches would require many technicians, an extraordinary number of resources, and is associated with significant product variability.

To enable parallel production of autologous iPSC-derived therapeutic cells, robotic technologies that can perform critical tasks largely unsupervised are required. Recently, several automated platforms designed to support iPSC generation, culture and differentiation have been developed. For instance, Tristan et al. described development of the CompacT SelecT (CTST) platform, which incorporates a robotic arm, liquid handling device, microscope, cell counter and multi-plate automated incubator [114]. Using this system the authors report automated culture, passage, and differentiation of up to 90 cell lines in parallel [114]. Using a similar strategy, we recently developed a robotic platform known as the CellX, which has the capability of automating iPSC generation [115]. Like the CTST, the CellX contains an automated microscope, plate mover and liquid handling capabilities [115]. By housing this system inside of a BiosSpherix Xvivo isolator, cultures can be maintained under reduced oxygen tension, which enhances iPSC reprogramming efficiency [116]. In addition, the CellX also contains a syringe pump, which uses sterile micropipette tips to pick iPSCs for clonal expansion following reprogramming and CRISPR correction as well as remove areas of spontaneous differentiation [115]. By incorporating image analysis algorithms into automated systems such as these, going forward it will be possible to monitor the cell product at each stage of development. For instance, in a recent study Schaub et al. reported the development of an image-based AI strategy for monitoring cellular maturation and demonstrating graft identity and function prior to transplantation [117]. This approach will enable nondestructive clinical release testing, greatly reducing the amount of product required for each patient, subsequently enhancing translatability of the autologous cell replacement approach.

### Dr. George Harb: “Scaled up and scaled out manufacturing strategies for ocular cell therapies”

Private, government and academic groups are investing in manufacturing technologies at scales fit for a range of cell therapy doses and patient population sizes. Current iPSC-based ocular cell therapies for degenerative eye diseases are manufactured using both scaled out and scaled up approaches.

Scaled out (decentralized) manufacturing platforms produce autologous cell therapies on a per patient basis. Automated, closed systems for parallel production, in lower-grade cleanrooms (Grade C) with in-process, noninvasive monitoring and feedback managed by artificial intelligence (AI). Cartridge, ‘cell-in-a-box’ and closed-cassette models are in development by Lonza, Hitachi, Cellares, and Cellino Biotech. Enabling next generation technologies in quantitative imaging, analytics and cell biology are combined with machine learning algorithms and omics datasets to improve overall cell therapy manufacturing fidelity. Features of iPSCs extracted from label-free morphological analyses include donor cell morphology, iPSC colony confluence, fate prediction, and differentiated iPSC-derived RPE product potency [117].

Development of end-to-end closed system processes with machine learning tracking of cell therapies is a strategic goal for several cell therapy approaches. Retinal pigmented epithelial cells (RPEs) are an ideal cell type for closed cassette manufacturing due to a relatively low 100,000 s cell dose to treat age-related macular degeneration and efficient differentiation production processes. As part of an ongoing Phase 1/2a clinical trial, iPSC-RPE cell therapies are in development by a collaborative effort between the National Eye Institute (NEI), FUJIFILM Cellular Dynamics Inc., and Opsis Therapeutics. The autologous iPSC-RPE cell therapy was developed using a clinical-grade manufacturing adherent process performed at the Center for Cellular Engineering (CCE) Clinical Center, National Institutes of Health (NIH) [6]. Manufactured iPSC-RPE products are in development as both adherent and suspension-based cell formulations. Adherent production platforms (see examples below) include the Hitachi ACE3, a closed, automated cell culture system with integrated monitoring. Suspension iPSC-RPE formulations are being produced in stirred-tank bioreactors including the Eppendorf BioBlu (3L) bioreactor. Suspension bioreactors are suitable for batch manufacturing allogeneic cell therapies and large cell therapy dose products in the hundreds of millions to billions of cells. *For hiPSC the media can use either a microcarrier (e.g., Pall Corporation collagen-coated) or since iPSCs like to grow in aggregates they can be grown as free aggregates (without microcarriers).* Single use vessels, tanks, or bags are available in stirred tank, vertical wheel geometries, and as hollow fiber platforms from 0.5L to 2000L scale.

**Table:** Features of an adherent, automated closed-culture system for autologous iPSC-RPE vs. a suspension-based bioreactors for off the shelf iPSC-RPE

	Riken-Hitachi	Lineage cell therapeutics
Cell source	Autologous iPSC-RPE cell therapy product	Allogenic (“off-the-shelf”) iPSC-RPE cell therapy product
Application	Adherent-based differentiation	Suspension-based differentiation
Bioprocess culture system employed	iPSC-RPE sheets machine-cultured using the Hitachi ACE3 automated cell culture system [96]	Scaled OpRegen manufacturing in Eppendorf BioBlu (3L) bioreactors
Total cell culturing area	Approximately 10^7^ cells per 42 cm^2^	5 billion cells per 3-L bioreactor
Opportunities for further scale-up	Can accommodate 10 culturing vessels	Further scale-up in larger reactors or scale-out in parallel reactors
Economic comparison	Replaces manual work and variable quality with machines	Immense process cost, labor, complexity
Institute	Kobe Eye Center	Lineage Cell Therapeutics

The next wave of innovations in cell therapy manufacturing will accelerate digital transformations, improve overall efficiency, and drive down costs of a single cell product from $440,000 USD as of summer 2021 (average of five approved cell therapies including autologous and allogenic cell sources) [118].

## Looking ahead

As described by the panel of speakers above, several factors including selection of starting cell source, manufacturing scalability, quality control, and immune response to the transplant are challenges that need to be addressed to transition a cell therapy product to clinical care. In addition, a hurdle encountered by many academic laboratories is the inability to test their newly developed therapies and conduct initial feasibility clinical testing, which precludes the formation of partnerships with biotechnology and pharmaceutical companies. For a cell-based product to transition to a therapy, there needs to be a rigorous and exacting approach that identifies the ideal cell product under specific conditions. It is not yet known which is more effective: allogeneic or autologous cell therapies, delivery of cell suspensions or scaffold-based tissue engineered approaches, whether support cells need to be transplanted as well, and whether there is—or ever will be—a universally effective treatment. This paucity of information is due to the lack of rigorous, head-to-head comparisons of various cell therapies. Additionally, markers to distinguish healthy from disease-laden cells for transplantation are not fully developed, limiting potential for automation and AI in quality control monitoring. Moreover, best practices for sharing stem cell lines, appropriate standards, and protocol validation have yet to be implemented and widely adopted.

Going forward, it will be necessary to identify opportunities that would advance cell replacement therapies for retinal disease. This includes developing unique intellectual property for cell therapy manufacturing, scalability, and delivery, facilitating partnerships with the private sector. Additionally, finding ways to enable academic laboratories to conduct feasibility testing and form partnerships with industry is necessary. There are industry partners from other agencies that specialize in working with academic laboratories that have a product but need assistance with manufacturing and scalability; this requires individuals or organizations working as scientific brokers to identify potential partnerships. Organizations such as the Advanced Regenerative Manufacturing Institute (ARMI) and the Alliance for Manufacturing Foresight (MFORESIGHT) work to facilitate these partnerships and provide resources to advance R&D across disciplines. At the government level, there are opportunities for investigators to obtain assistance with product development as well. The FDA has the office of Tissues and Advanced Therapies, an office of the Center for Biologics Evaluation and Research (CBER). The NIH provides grant opportunities to help develop early-stage research and commercialize new translational technologies through the Small Business Innovation Research (SBIR) and Small Business Technology Transfer (STTR) Programs. Moreover, it will be important to continue to recognize the value of basic research and exploratory studies focused on rigorously comparing cell-based products in various host conditions and identifying markers to indicate when cells are amenable for transplantation to facilitate automated processing.

## Data Availability

Not applicable.

## Abbreviations

ARVO: Association for Research in Vision and Ophthalmology
NEI: National Eye Institute
RPE: Retinal Pigment Epithelium
iPSCs: Induced pluripotent stem cells
hESCs: Human embryonic stem cells
AMD: Age-related macular degeneration
HLA: Human leukocyte antigen
RPESC: Adult RPE stem cell
QC: Quality control
CQA: Critical quality attribute
MHC: Major histocompatibility complex
ACAID: Anterior chamber-associated immunity deviation
AI: Artificial intelligence
IND: Investigational new drug
CRISPR: Clustered Regularly Interspaced Short Palindromic Repeats
RNA: Ribonucleic acid
DNA: Deoxyribonucleic acid
CD4: Cluster of differentiation 4
CD8: Cluster of differentiation 8
CTST: CompacT SelecT
EU: European Union
UK: United Kingdom
hPSC: Human pluripotent stem cell
CCE: Center for Cellular Engineering
USD: United States Dollar
ARMI: Advanced Regenerative Manufacturing Institute
MFORESIGHT: Alliance for Manufacturing Foresight
FDA: US Food and Drug Administration
CBER: Center for Biologics Evaluation and Research
SBIR: Small Business Innovation Research
STTR: Small Business Technology Transfer

## References

[1] National Eye Institute. National Institutes of Health Strategic Planning [cited 2022 August 15]. https://www.nei.nih.gov/about/strategic-planning.

[2] Chiang MF, Tumminia SJ (2022). The 2021 National Eye Institute Strategic Plan: eliminating vision loss and improving quality of life. Ophthalmology.

[3] Garreta E, Sanchez S, Lajara J, Montserrat N, Belmonte JCI (2018). Roadblocks in the path of iPSC to the clinic. Curr Transpl Rep.

[4] Kashani AH, Lebkowski JS, Rahhal FM, Avery RL, Salehi-Had H, Dang W (2018). A bioengineered retinal pigment epithelial monolayer for advanced, dry age-related macular degeneration. Sci Transl Med..

[5] Saini JS, Temple S, Stern JH (2016). Human retinal pigment epithelium stem cell (RPESC). Adv Exp Med Biol.

[6] Sharma R, Khristov V, Rising A, Jha BS, Dejene R, Hotaling N (2019). Clinical-grade stem cell-derived retinal pigment epithelium patch rescues retinal degeneration in rodents and pigs. Sci Transl Med..

[7] Sharma R, Bose D, Maminishkis A, Bharti K (2020). Retinal pigment epithelium replacement therapy for age-related macular degeneration: are we there yet?. Annu Rev Pharmacol Toxicol.

[8] Schwartz SD, Hubschman JP, Heilwell G, Franco-Cardenas V, Pan CK, Ostrick RM (2012). Embryonic stem cell trials for macular degeneration: a preliminary report. Lancet.

[9] Keenan TDL, Cukras CA, Chew EY (2021). Age-related macular degeneration: epidemiology and clinical aspects. Adv Exp Med Biol.

[10] Sieving PA (2012). NEI audacious goals initiative to catalyze innovation. Invest Ophthalmol Vis Sci.

[11] Salowe RJ, O'Brien JM (2014). NEI's audacious goals initiative. Ophthalmology.

[12] Zhou R, Caspi RR (2010). Ocular immune privilege. F1000 Biol Rep..

[13] Taylor AW (2016). Ocular immune privilege and transplantation. Front Immunol.

[14] Singh MS, Park SS, Albini TA, Canto-Soler MV, Klassen H, MacLaren RE (2020). Retinal stem cell transplantation: balancing safety and potential. Prog Retin Eye Res.

[15] Williams DR (2011). Imaging single cells in the living retina. Vis Res.

[16] Liu T, Aguilera N, Bower AJ, Li J, Ullah E, Dubra A (2022). Photoreceptor and retinal pigment epithelium relationships in eyes with vitelliform macular dystrophy revealed by multimodal adaptive optics imaging. Invest Ophthalmol Vis Sci.

[17] Sugita S, Mandai M, Kamao H, Takahashi M (2021). Immunological aspects of RPE cell transplantation. Prog Retin Eye Res.

[18] Fujii S, Sugita S, Futatsugi Y, Ishida M, Edo A, Makabe K (2020). A strategy for personalized treatment of iPS-retinal immune rejections assessed in cynomolgus monkey models. Int J Mol Sci.

[19] Miyagishima KJ, Wan Q, Corneo B, Sharma R, Lotfi MR, Boles NC (2016). In pursuit of authenticity: induced pluripotent stem cell-derived retinal pigment epithelium for clinical applications. Stem Cells Transl Med.

[20] Miyagishima KJ, Wan Q, Miller SS, Bharti K (2017). A basis for comparison: sensitive authentication of stem cell derived RPE using physiological responses of intact RPE monolayers. Stem Cell Transl Investig.

[21] Thomson JA, Itskovitz-Eldor J, Shapiro SS, Waknitz MA, Swiergiel JJ, Marshall VS (1998). Embryonic stem cell lines derived from human blastocysts. Science.

[22] Mehat MS, Sundaram V, Ripamonti C, Robson AG, Smith AJ, Borooah S (2018). Transplantation of human embryonic stem cell-derived retinal pigment epithelial cells in macular degeneration. Ophthalmology.

[23] Li SY, Liu Y, Wang L, Wang F, Zhao TT, Li QY (2021). A phase I clinical trial of human embryonic stem cell-derived retinal pigment epithelial cells for early-stage Stargardt macular degeneration: 5-years' follow-up. Cell Prolif.

[24] Schwartz SD, Tan G, Hosseini H, Nagiel A (2016). Subretinal transplantation of embryonic stem cell-derived retinal pigment epithelium for the treatment of macular degeneration: an assessment at 4 years. Invest Ophthalmol Vis Sci.

[25] Kashani AH, Lebkowski JS, Rahhal FM, Avery RL, Salehi-Had H, Chen S (2021). One-year follow-up in a phase 1/2a clinical trial of an allogeneic RPE cell bioengineered implant for advanced dry age-related macular degeneration. Transl Vis Sci Technol.

[26] da Cruz L, Fynes K, Georgiadis O, Kerby J, Luo YH, Ahmado A (2018). Phase 1 clinical study of an embryonic stem cell-derived retinal pigment epithelium patch in age-related macular degeneration. Nat Biotechnol.

[27] da Tsokas K, McFarland R, Burke C, Lynch JL, Bollenbach T, Callaway DA (2019). Reducing risks and delays in the translation of cell and gene therapy innovations into regulated products. NAM Perspect.

[28] Liu Z, Parikh BH, Tan QSW, Wong DSL, Ong KH, Yu W (2021). Surgical transplantation of human RPE stem cell-derived RPE monolayers into non-human primates with immunosuppression. Stem Cell Rep.

[29] May-Simera HL, Wan Q, Jha BS, Hartford J, Khristov V, Dejene R (2018). Primary cilium-mediated retinal pigment epithelium maturation is disrupted in ciliopathy patient cells. Cell Rep.

[30] Dashnau JL, Xue Q, Nelson M, Law E, Cao L, Hei D (2023). A risk-based approach for cell line development, manufacturing and characterization of genetically engineered, induced pluripotent stem cell-derived allogeneic cell therapies. Cytotherapy.

[31] Khristov V, Wan Q, Sharma R, Lotfi M, Maminishkis A, Bharti K (2018). Polarized human retinal pigment epithelium exhibits distinct surface proteome on apical and basal plasma membranes. Methods Mol Biol.

[32] Diniz B, Thomas P, Thomas B, Ribeiro R, Hu Y, Brant R (2013). Subretinal implantation of retinal pigment epithelial cells derived from human embryonic stem cells: improved survival when implanted as a monolayer. Invest Ophthalmol Vis Sci.

[33] Kamao H, Mandai M, Ohashi W, Hirami Y, Kurimoto Y, Kiryu J (2017). Evaluation of the surgical device and procedure for extracellular matrix-scaffold-supported human iPSC-derived retinal pigment epithelium cell sheet transplantation. Invest Ophthalmol Vis Sci.

[34] Li KV, Flores-Bellver M, Aparicio-Domingo S, Petrash C, Cobb H, Chen C (2022). A surgical kit for stem cell-derived retinal pigment epithelium transplants: collection, transportation, and subretinal delivery. Front Cell Dev Biol.

[35] Lytvynchuk L, Ebbert A, Studenovska H, Nagymihály R, Josifovska N, Rais D (2022). Subretinal implantation of human primary RPE cells cultured on nanofibrous membranes in minipigs. Biomedicines.

[36] Nishida M, Tanaka Y, Amaya S, Tanaka N, Uyama H, Masuda T (2021). Human iPS cell derived RPE strips for secure delivery of graft cells at a target place with minimal surgical invasion. Sci Rep.

[37] Alexander P, Thomson HA, Luff AJ, Lotery AJ (2015). Retinal pigment epithelium transplantation: concepts, challenges, and future prospects. Eye (London).

[38] Petrus-Reurer S, Winblad N, Kumar P, Gorchs L, Chrobok M, Wagner AK (2020). Generation of retinal pigment epithelial cells derived from human embryonic stem cells lacking human leukocyte antigen class I and II. Stem Cell Rep.

[39] Jang Y, Choi J, Park N, Kang J, Kim M, Kim Y (2019). Development of immunocompatible pluripotent stem cells via CRISPR-based human leukocyte antigen engineering. Exp Mol Med.

[40] Takahashi K, Yamanaka S (2006). Induction of pluripotent stem cells from mouse embryonic and adult fibroblast cultures by defined factors. Cell.

[41] Fusaki N, Ban H, Nishiyama A, Saeki K, Hasegawa M (2009). Efficient induction of transgene-free human pluripotent stem cells using a vector based on Sendai virus, an RNA virus that does not integrate into the host genome. Proc Jpn Acad Ser B Phys Biol Sci.

[42] Febbraro F, Chen M, Denham M (2021). Generation of human iPSCs by episomal reprogramming of skin fibroblasts and peripheral blood mononuclear cells. Methods Mol Biol.

[43] Warren L, Lin C (2019). mRNA-based genetic reprogramming. Mol Ther.

[44] Biswas D, Jiang P (2016). Chemically induced reprogramming of somatic cells to pluripotent stem cells and neural cells. Int J Mol Sci.

[45] Jia F, Wilson KD, Sun N, Gupta DM, Huang M, Li Z (2010). A nonviral minicircle vector for deriving human iPS cells. Nat Methods.

[46] Woltjen K, Michael IP, Mohseni P, Desai R, Mileikovsky M, Hämäläinen R (2009). piggyBac transposition reprograms fibroblasts to induced pluripotent stem cells. Nature.

[47] Seo BJ, Hong YJ, Do JT (2017). Cellular reprogramming using protein and cell-penetrating peptides. Int J Mol Sci.

[48] Guan J, Wang G, Wang J, Zhang Z, Fu Y, Cheng L (2022). Chemical reprogramming of human somatic cells to pluripotent stem cells. Nature.

[49] Takagi S, Mandai M, Gocho K, Hirami Y, Yamamoto M, Fujihara M (2019). Evaluation of transplanted autologous induced pluripotent stem cell-derived retinal pigment epithelium in exudative age-related macular degeneration. Ophthalmol Retina.

[50] Taylor AW, Hsu S, Ng TF (2021). The role of retinal pigment epithelial cells in regulation of macrophages/microglial cells in retinal immunobiology. Front Immunol.

[51] Idelson M, Alper R, Obolensky A, Yachimovich-Cohen N, Rachmilewitz J, Ejzenberg A (2018). Immunological properties of human embryonic stem cell-derived retinal pigment epithelial cells. Stem Cell Rep.

[52] Ripolles-Garcia A, Dolgova N, Phillips MJ, Savina S, Ludwig AL, Stuedemann SA (2022). Systemic immunosuppression promotes survival and integration of subretinally implanted human ESC-derived photoreceptor precursors in dogs. Stem Cell Rep.

[53] Doulgkeroglou MN, Di Nubila A, Niessing B, König N, Schmitt RH, Damen J (2020). Automation, monitoring, and standardization of cell product manufacturing. Front Bioeng Biotechnol.

[54] Aneesh A, Liu A, Moss HE, Feinstein D, Ravindran S, Mathew B (2021). Emerging concepts in the treatment of optic neuritis: mesenchymal stem cell-derived extracellular vesicles. Stem Cell Res Ther.

[55] Mead B, Tomarev S (2017). Bone marrow-derived mesenchymal stem cells-derived exosomes promote survival of retinal ganglion cells through miRNA-dependent mechanisms. Stem Cells Transl Med.

[56] Zhang W, Wang Y, Kong Y (2019). Exosomes derived from mesenchymal stem cells modulate miR-126 to ameliorate hyperglycemia-induced retinal inflammation via targeting HMGB1. Invest Ophthalmol Vis Sci.

[57] Usategui-Martín R, Puertas-Neyra K, Galindo-Cabello N, Hernández-Rodríguez LA, González-Pérez F, Rodríguez-Cabello JC (2022). Retinal neuroprotective effect of mesenchymal stem cells secretome through modulation of oxidative stress, autophagy, and programmed cell death. Invest Ophthalmol Vis Sci.

[58] Bin Imtiaz MK, Jaeger BN, Bottes S, Machado RAC, Vidmar M, Moore DL (2021). Declining lamin B1 expression mediates age-dependent decreases of hippocampal stem cell activity. Cell Stem Cell.

[59] Aladdad AM, Kador KE (2019). Adult stem cells, tools for repairing the retina. Curr Ophthalmol Rep.

[60] Gu P, Harwood LJ, Zhang X, Wylie M, Curry WJ, Cogliati T (2007). Isolation of retinal progenitor and stem cells from the porcine eye. Mol Vis.

[61] Courtney JM, Sutherland BA (2020). Harnessing the stem cell properties of pericytes to repair the brain. Neural Regen Res.

[62] Abbas O, Mahalingam M (2009). Epidermal stem cells: practical perspectives and potential uses. Br J Dermatol.

[63] Hassan HT, El-Sheemy M (2004). Adult bone-marrow stem cells and their potential in medicine. J R Soc Med.

[64] Schmidt M, Schüler SC, Hüttner SS, von Eyss B, von Maltzahn J (2019). Adult stem cells at work: regenerating skeletal muscle. Cell Mol Life Sci.

[65] Aldahmash A, Zaher W, Al-Nbaheen M, Kassem M (2012). Human stromal (mesenchymal) stem cells: basic biology and current clinical use for tissue regeneration. Ann Saudi Med.

[66] Seale P, Asakura A, Rudnicki MA (2001). The potential of muscle stem cells. Dev Cell.

[67] Sullivan S, Stacey GN, Akazawa C, Aoyama N, Baptista R, Bedford P (2018). Quality control guidelines for clinical-grade human induced pluripotent stem cell lines. Regen Med.

[68] Jha BS, Farnoodian M, Bharti K (2021). Regulatory considerations for developing a phase I investigational new drug application for autologous induced pluripotent stem cells-based therapy product. Stem Cells Transl Med.

[69] Salero E, Blenkinsop TA, Corneo B, Harris A, Rabin D, Stern JH (2012). Adult human RPE can be activated into a multipotent stem cell that produces mesenchymal derivatives. Cell Stem Cell.

[70] Blenkinsop TA, Saini JS, Maminishkis A, Bharti K, Wan Q, Banzon T (2015). Human adult retinal pigment epithelial stem cell-derived RPE monolayers exhibit key physiological characteristics of native tissue. Invest Ophthalmol Vis Sci.

[71] Davis RJ, Blenkinsop TA, Campbell M, Borden SM, Charniga CJ, Lederman PL (2016). Human RPE stem cell-derived rpe preserves photoreceptors in the Royal College Of Surgeons rat: method for quantifying the area of photoreceptor sparing. J Ocul Pharmacol Ther.

[72] Davis RJ, Alam NM, Zhao C, Müller C, Saini JS, Blenkinsop TA (2017). The developmental stage of adult human stem cell-derived retinal pigment epithelium cells influences transplant efficacy for vision rescue. Stem Cell Rep.

[73] Safety and Tolerability of RPESC-derived RPE Transplantation in Patients With Dry Age-related Macular Degeneration (AMD). https://clinicaltrials.gov/ct2/show/NCT04627428. 10.1016/j.stem.2025.08.012 PMC12533590 40961946

[74] Lingam S, Liu Z, Yang B, Wong W, Parikh BH, Ong JY (2021). cGMP-grade human iPSC-derived retinal photoreceptor precursor cells rescue cone photoreceptor damage in non-human primates. Stem Cell Res Ther.

[75] Gasparini SJ, Tessmer K, Reh M, Wieneke S, Carido M, Völkner M (2022). Transplanted human cones incorporate into the retina and function in a murine cone degeneration model. J Clin Invest.

[76] Lamba DA, McUsic A, Hirata RK, Wang PR, Russell D, Reh TA (2010). Generation, purification and transplantation of photoreceptors derived from human induced pluripotent stem cells. PLoS ONE.

[77] Zhang X, Tenerelli K, Wu S, Xia X, Yokota S, Sun C (2020). Cell transplantation of retinal ganglion cells derived from hESCs. Restor Neurol Neurosci.

[78] Kupperman BD, Boyer DS, Mills B, Yang J, Klassen (2018). Safety and activity of a single, intravitreal injection of human retinal progenitor cells (jCell) for treament of retinitis pigmentosa. Invest Ophthalmol Vis Sci.

[79] Turner L, Knoepfler P (2016). Selling stem cells in the USA: assessing the direct-to-consumer industry. Cell Stem Cell.

[80] Kuriyan AE, Albini TA, Townsend JH, Rodriguez M, Pandya HK, Leonard RE (2017). Vision loss after intravitreal injection of autologous "stem cells" for AMD. N Engl J Med.

[81] Chavez J, Shah NA, Ruoss S, Cuomo RE, Ward SR, Mackey TK (2021). Online marketing practices of regenerative medicine clinics in US-Mexico border region: a web surveillance study. Stem Cell Res Ther.

[82] Orozco-Solares TE, León-Moreno LC, Rojas-Rizo A, Manguart-Páez K, Caplan AI (2022). Allogeneic mesenchymal stem cell-based treatment legislation in Latin America: the need for standardization in a medical tourism context. Stem Cells Dev.

[83] Lyons S, Salgaonkar S, Flaherty GT (2022). International stem cell tourism: a critical literature review and evidence-based recommendations. Int Health.

[84] Ogbogu U, Du L, Rachul C, Bélanger L, Caulfield T (2013). Chinese newspaper coverage of (unproven) stem cell therapies and their providers. Stem Cell Rev Rep.

[85] Lv J, Su Y, Song L, Gong X, Peng Y (2020). Stem cell 'therapy' advertisements in China: infodemic, regulations and recommendations. Cell Prolif.

[86] Rosemann A, Sleeboom-Faulkner M (2016). New regulation for clinical stem cell research in China: expected impact and challenges for implementation. Regen Med.

[87] Li C (2022). Strengthening regulations, recent advances and remaining barriers in stem cell clinical translation in China: 2015–2021 in review. Pharmacol Res.

[88] Gao J, Gao C (2022). Development and regulation of stem cell-based therapies in China. Cell Prolif.

[89] Nine Things to Know About Stem Cell Treatments. https://www.closerlookatstemcells.org/stem-cells-medicine/nine-things-to-know-about-stem-cell-treatments.

[90] Abranches E, Spyrou S, Ludwig T (2020). GMP banking of human pluripotent stem cells: a US and UK perspective. Stem Cell Res.

[91] Medawar PB (1948). Immunity to homologous grafted skin; the fate of skin homografts transplanted to the brain, to subcutaneous tissue, and to the anterior chamber of the eye. Br J Exp Pathol.

[92] Forrester JV, Xu H (2012). Good news-bad news: the Yin and Yang of immune privilege in the eye. Front Immunol.

[93] Petrash CC, Palestine AG, Canto-Soler MV (2021). Immunologic rejection of transplanted retinal pigmented epithelium: mechanisms and strategies for prevention. Front Immunol.

[94] Kashani AH, Lebkowski JS, Hinton DR, Zhu D, Faynus MA, Chen S (2022). Survival of an HLA-mismatched, bioengineered RPE implant in dry age-related macular degeneration. Stem Cell Rep.

[95] McGill TJ, Stoddard J, Renner LM, Messaoudi I, Bharti K, Mitalipov S (2018). Allogeneic iPSC-derived RPE cell graft failure following transplantation into the subretinal space in nonhuman primates. Invest Ophthalmol Vis Sci.

[96] Ruan GP, Yao X, Liu JF, He J, Li ZA, Yang JY (2016). Establishing a tree shrew model of systemic lupus erythematosus and cell transplantation treatment. Stem Cell Res Ther.

[97] Gouras P, Du J, Kjeldbye H, Yamamoto S, Zack DJ (1994). Long-term photoreceptor transplants in dystrophic and normal mouse retina. Invest Ophthalmol Vis Sci.

[98] Yu CT, Kandoi S, Periasamy R, Follett HM, Summerfelt P, Guillaume C (2022). Exploring the survival of transplanted hiPSC-derived photoreceptors in the 13-lined ground squirrel. Invest Ophthalmol Vis Sci.

[99] Nelson J, Alvey N, Bowman L, Schulte J, Segovia MC, McDermott J (2022). Consensus recommendations for use of maintenance immunosuppression in solid organ transplantation: endorsed by the American College of Clinical Pharmacy, American Society of Transplantation, and the International Society for Heart and Lung Transplantation. Pharmacotherapy.

[100] Chan YK, Wang SK, Chu CJ, Copland DA, Letizia AJ, Costa Verdera H (2021). Engineering adeno-associated viral vectors to evade innate immune and inflammatory responses. Sci Transl Med..

[101] Petrus-Reurer S, Romano M, Howlett S, Jones JL, Lombardi G, Saeb-Parsy K (2021). Immunological considerations and challenges for regenerative cellular therapies. Commun Biol.

[102] National Academies of Sciences Eg, and Medicine, Division HaM, Policy BoHS, Medicine FoR. Understanding the role of the immune system in improving tissue regeneration: proceedings of a workshop. 2022. 35467816

[103] Raulet DH (2006). Missing self recognition and self tolerance of natural killer (NK) cells. Semin Immunol.

[104] Markert EK, Klein H, Viollet C, Rust W, Strobel B, Kauschke SG (2022). Transcriptional comparison of adult human primary Retinal Pigment Epithelium, human pluripotent stem cell-derived Retinal Pigment Epithelium, and ARPE19 cells. Front Cell Dev Biol.

[105] Mayshar Y, Ben-David U, Lavon N, Biancotti JC, Yakir B, Clark AT (2010). Identification and classification of chromosomal aberrations in human induced pluripotent stem cells. Cell Stem Cell.

[106] Martins-Taylor K, Nisler BS, Taapken SM, Compton T, Crandall L, Montgomery KD (2011). Recurrent copy number variations in human induced pluripotent stem cells. Nat Biotechnol.

[107] Merkle FT, Ghosh S, Kamitaki N, Mitchell J, Avior Y, Mello C (2017). Human pluripotent stem cells recurrently acquire and expand dominant negative P53 mutations. Nature.

[108] Jinek M, East A, Cheng A, Lin S, Ma E, Doudna J (2013). RNA-programmed genome editing in human cells. Elife.

[109] Burnight ER, Gupta M, Wiley LA, Anfinson KR, Tran A, Triboulet R (2017). Using CRISPR-Cas9 to generate gene-corrected autologous iPSCs for the treatment of inherited retinal degeneration. Mol Ther.

[110] Burnight ER, Bohrer LR, Giacalone JC, Klaahsen DL, Daggett HT, East JS (2018). CRISPR-Cas9-mediated correction of the 1.02 kb common deletion in. CRISPR J..

[111] Bohrer LR, Wiley LA, Burnight ER, Cooke JA, Giacalone JC, Anfinson KR (2019). Correction of NR2E3 associated enhanced S-cone syndrome patient-specific iPSCs using CRISPR-Cas9. Genes (Basel).

[112] Weisheit I, Kroeger JA, Malik R, Klimmt J, Crusius D, Dannert A (2020). Detection of Deleterious on-target effects after HDR-mediated CRISPR editing. Cell Rep.

[113] Simkin D, Papakis V, Bustos BI, Ambrosi CM, Ryan SJ, Baru V (2022). Homozygous might be hemizygous: CRISPR/Cas9 editing in iPSCs results in detrimental on-target defects that escape standard quality controls. Stem Cell Rep.

[114] Tristan CA, Ormanoglu P, Slamecka J, Malley C, Chu PH, Jovanovic VM (2021). Robotic high-throughput biomanufacturing and functional differentiation of human pluripotent stem cells. Stem Cell Rep.

[115] Mullin NK, Voigt AP, Cooke JA, Bohrer LR, Burnight ER, Stone EM (2021). Patient derived stem cells for discovery and validation of novel pathogenic variants in inherited retinal disease. Prog Retin Eye Res.

[116] Yoshida Y, Takahashi K, Okita K, Ichisaka T, Yamanaka S (2009). Hypoxia enhances the generation of induced pluripotent stem cells. Cell Stem Cell.

[117] Schaub NJ, Hotaling NA, Manescu P, Padi S, Wan Q, Sharma R (2020). Deep learning predicts function of live retinal pigment epithelium from quantitative microscopy. J Clin Invest.

[118] Whitby P. Cell & gene therapy: high stakes and high hopes for healthcare 2022. https://www.reutersevents.com/pharma/commercial/cell-gene-therapy-high-stakes-and-high-hopes-healthcare.

